# Virus wars: using one virus to block the spread of another

**DOI:** 10.7717/peerj.2166

**Published:** 2016-06-29

**Authors:** Matthew L. Paff, Scott L. Nuismer, Andrew Ellington, Ian J. Molineux, James J. Bull

**Affiliations:** 1 Department of Integrative Biology, University of Texas, Austin, TX, United States; 2 Department of Biological Sciences, University of Idaho, Moscow, ID, United States; 3 Department of Molecular Biosciences, University of Texas at Austin, Austin, TX, United States; 4 The Institute for Cellular and Molecular Biology, University of Texas, Austin, TX, United States; 5 Center for Systems, Biology, University of Texas at Austin, Austin, TX, United States; 6 Center for Computational Biology and Bioinformatics, University of Texas, Austin, TX, United States

**Keywords:** Infectious vaccine, Gene therapy, Population dynamics, Vaccine alternative, Intervention, Bacteriophage, Mathematical model

## Abstract

The failure of traditional interventions to block and cure HIV infections has led to novel proposals that involve treating infections with therapeutic viruses–infectious viruses that specifically inhibit HIV propagation in the host. Early efforts in evaluating these proposals have been limited chiefly to mathematical models of dynamics, for lack of suitable empirical systems. Here we propose, develop and analyze an empirical system of a therapeutic virus that protects a host cell population against a lethal virus. The empirical system uses *E. coli* bacteria as the host cell population, an RNA phage as the lethal virus and a filamentous phage as the therapeutic virus. Basic dynamic properties are established for each virus alone and then together. Observed dynamics broadly agree with those predicted by a computer simulation model, although some differences are noted. Two cases of dynamics are contrasted, differing in whether the therapeutic virus is introduced before the lethal virus or after the lethal virus. The therapeutic virus increases in both cases but by different mechanisms. With the therapeutic virus introduced first, it spreads infectiously without any appreciable change in host dynamics. With the therapeutic virus introduced second, host abundance is depressed at the time therapy is applied; following an initial period of therapeutic virus spread by infection, the subsequent rise of protection is through reproduction by hosts already protected. This latter outcome is due to inheritance of the therapeutic virus state when the protected cell divides. Overall, the work establishes the feasibility and robustness to details of a viral interference using a therapeutic virus.

## Introduction

HIV-1 infections dodge many of the standard medical interventions. Thus, a vaccine remains elusive, and drugs suppress viral growth within the person but do not cure because of latent infections in cells. These failures have inspired proposals of novel interventions that take advantage of the long term, chronic nature of HIV-1 infections. Several proposals have in common a form of dynamical suppression akin to a ‘virus war,’ whereby a therapeutic virus is introduced into the patient to dynamically limit the number of HIV-infected cells and thereby prevent collapse of the immune system. In one proposal, the therapeutic virus specifically infects and kills HIV-infected cells, and viruses have even been engineered with the appropriate tropisms (Schnell et al., 1997; Mebatsion et al., 1997). In another, the therapeutic virus is an infectious, genomic ‘parasite’ that suppresses HIV-1 reproduction when both infect the same cell (Weinberger, Schaffer & Arkin, 2003; Metzger, Lloyd-Smith & Weinberger, 2011; Ke & Lloyd-Smith, 2012). A third approach is to genetically convert the target cells to resist HIV infection altogether, either by infusing the patient with stem cells converted to resistance *in vitro* (Leonard & Schaffer, 2005; Pandit & De Boer, 2015; Petit & Marodon, 2016) or by introducing a protective virus that spreads within the patient.

One obvious challenge confronting the success of each approach is obtaining a therapeutic agent that performs the required task at the cellular level, whether to kill an HIV-infected cell, suppress HIV reproduction, or convert the cell to resistance. Genetic engineering now renders those tasks within reach. An equally important requirement, and one often overlooked, is suitable dynamics at the population level. The simple fact that one transmissible agent kills or interferes with another is not sufficient to ensure that the interference will have much impact at the population level. Sophisticated mathematical analyses have recently begun to explore the feasibility of dynamic suppression approaches against HIV-1 (Weinberger, Schaffer & Arkin, 2003; Metzger, Lloyd-Smith & Weinberger, 2011; Ke & Lloyd-Smith, 2012), but there remains a dearth of empirical studies to explore the vulnerabilities of those models. Here we explore one approach to dynamic suppression in a model system using bacteriophages. Our system is dynamically equivalent to transmissible gene therapy to induce cellular resistance against a lethal virus, although the underlying mechanism is not strictly one of gene therapy. The biology of our system is distinct from HIV-1 infections, but its dynamics closely mimic those used to model dynamic suppression of HIV-1 (Weinberger, Schaffer & Arkin, 2003; Metzger, Lloyd-Smith & Weinberger, 2011). The system has the advantage of being far more empirically tractable than more realistic alternatives, allowing us to discover possible shortcomings and challenges of dynamic suppression model approaches.

### The empirical system

We introduce and analyze an empirical system of two bacteriophages and a host cell in which a non-lethal virus protects the host from the other virus. The elements of our system are: a single bacterial host (*E. coli*), a lethal bacteriophage whose numbers we wish to limit, and a non-lethal phage as the therapeutic agent that protects host cells from the lethal bacteriophage. The following paragraphs describe the essential details of each entity.

#### The host: *E. coli* strain A/*λ*

*E. coli* is the typical host used for propagating the phages used here (Q*β* and f1), but to be infected by either phage, the host must harbor the F plasmid. We evaluated two different F-bearing *E. coli* for suitability as hosts for Q*β* infection (A/*λ* and IJ338) and chose the former because it was killed by Q*β* more rapidly and to lower levels than was IJ338. Even with A/*λ*, the dynamic profile exhibits a much higher minimum cell density than is typical or predicted in the presence of lytic phages (2–3 logs below the density in the absence of phage), a point we return to below. The ramifications of a high minimum cell density is that it limits the magnitude of any beneficial effect of an intervention.

#### The lethal virus: Q*β*

The phage Q*β* is used here to cause a lethal infection of its bacterial host. This phage has a genome of 4,127 bases of linear, single-stranded RNA with four protein coding genes. For the purposes of this study, the important attributes of this phage are twofold: (i) It is obligately lytic, so that an infection invariably leads to lysis and thus death of the cell 40–50 min after infection (lysis enables release of the Q*β* progeny) and (ii) it infects F-piliated *E. coli*; the side of the pilus is used as the receptor, and the phage genome enters through the pilus (Van Duin, 1988; Tsukada et al., 2009; Manchak, Anthony & Frost, 2002). The relevance of the Q*β* receptor (the F-pilus) will become apparent when discussing the therapeutic virus.

#### The therapeutic virus: f1

The filamentous phage f1 (nearly identical to the better known phage M13) also infects F-piliated *E. coli*; the tip of the pilus is used as the initial attachment site (its primary receptor), but retraction of the pilus brings it in contact with the secondary receptor, the tolA protein on the outer surface of the bacterium, from which infection occurs (Jacobson, 1972; Click & Webster, 1997). The f1 genome packaged in the viral particle is 6,407 bases of circular, single-stranded DNA, but upon infection, the ssDNA is converted to a dsDNA circle from which state all phage functions are expressed (Russel & Model, 2006; Marvin & Hohn, 1969). Eleven protein coding genes are recognized, and an intergenic region contains several regulatory signals and is also suitable for insertion of cloned sequences.

Phage f1 is atypical of most bacteriophages in that it does not lyse or kill its host; it establishes a persistent infection throughout the life of the host, and the infection is transmitted to daughter cells when the host divides–like a plasmid. Although f1 does not kill cells, it does adversely affect the growth rate and maximal cell density of the infected host (Table 1), and this growth reduction may be important to overall dynamics. Phage progeny production is via continual secretion of phage virions through the cell wall and membranes. Virion assembly occurs as the genome is extruded through the membranes. We measured the rate of phage output as 1.6/min (values 2.3, 0.98) from host A/*λ* (see Data S1).

**Table 1:** Properties of *E. coli* A/*λ* cells infected with f1.

Cell state	Intrinsic growth rate (h)	Maximal density^a^
Uninfected	1.98 ± 0.107	3.2 × 10^9^ ± 1.7 × 10^8^
Infected	1.62 ± 0.075	5.4 × 10^8^ ± 1.5 × 10^8^

**Notes.**

a Density after 18–24 h growth.

A *t*-test of the difference in maximal density is significant (*P* < 0.004, 2-tailed). A *t*-test of the difference in growth rates is marginally significant (*P* ≈ 0.03, 2-tailed).

**Figure 1:**
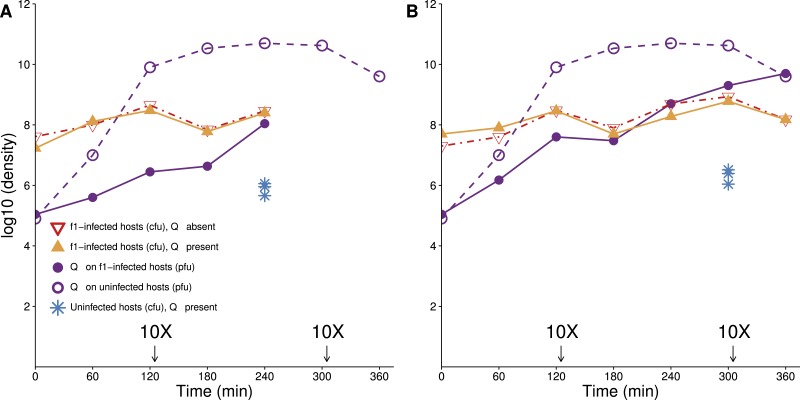
Lethal virus Q*β* growth on a population of f1-infected A/*λ* (A and B are independent replicates). Cells infected with therapeutic virus (phage f1) are largely resistant to infection by Q*β*, because their densities follow similar trajectories in the presence as in the absence of Q*β*. There is nonetheless some growth of Q*β* on these cells. A culture of cells infected with f1 carrying kanamycin resistance was grown overnight in LB with kanamycin (50 µg/mL). Cells were pelleted, and the pellet was re-suspended in 10 ml LB lacking drug and grown for 1 h before phage Q*β* was added to a concentration of 10^4^–10^5^/mL. To maintain cells in a continual state of growth (which enhances infection), 10× dilutions were made immediately after some platings, as indicated (densities are not adjusted for dilutions). Curves with triangles give densities of f1-infected cells, curves with circles give densities of lethal virus Q*β*. For comparison, the density of sensitive hosts in the presence of Q*β* alone is shown from time 240 (A) and 300 (B) (blue stars, from Fig. 3).

Several properties of f1 infections vary considerably, depending at least on the age of the infection and possibly on unknown variables (Shapiro, Williams & Turner, 2016; J Shapiro, pers. comm., 2016). In particular, the newly-infected cell must adjust to the rapid increase in viral genome copy number and to the sudden presence of phage proteins affecting membrane architecture. Thus, phage output from an infected cell will not be constant in time, and growth rate of the infected host is also apparently subject to various effects. Our model will not attempt to capture this complexity, instead relying on exploration of different parameter values to evaluate robustness.

An important property of infection by f1 is that within minutes of infection, the pilus is retracted or modified, so that infection by other filamentous phages is blocked (Palchoudhury & Iyer, 1969). The effect on the pilus has also been noted for blocking conjugation and biofilm formation (Lin et al., 2011; May, Tsuruta & Okabe, 2011). Pilus retraction protects the infecting f1 genome from being superinfected by other filamentous phage genomes that might usurp the resident genome, and we anticipated that infection by f1 would also block infection by other types of phages requiring the pilus. This expectation was supported with the observation that the titer of f1-infected cells was not noticeably depressed in the presence of Q*β* (Fig. 1). As the cells in this assay had been infected with f1 for at least 12 h before the challenge with Q*β*, these data do not inform as to how quickly after infection the host is protected, but it appears that the protection is not complete (or is slow). In theory, the mechanism of protection by f1 could be from retraction of the pilus or even from loss of the F plasmid. Use of a test using T7 (see Methods) on f1-infected A/*λ* grown overnight suggested that the F plasmid is retained in a large majority of cells, possibly all of them.

Overall, the protection from f1 evident from Fig. 1 is nearly two orders of magnitude (compare the yellow curve to the blue dots). The visual impression on a log plot is not necessarily impressive, but it amounts to a near 100-fold improvement in actual numbers. Furthermore, the protection afforded by f1 is nearly the maximum possible, because f1-infected cells do not achieve noticeably higher densities in the absence of the lethal phage as in its presence.

## Methods

### Strains and media

*Bacteria*. *E. coli* A/*λ* (Hirashima et al., 1986) was used for dynamics experiments. IJ338 (Messenger, Molineux & Bull, 1999) was also used in preliminary work.

*Media*. Bacteria and phages were cultured in LB broth (10 g NaCl, 10 g Bacto tryptone, 5 g Bacto yeast extract per liter). Plates contained LB with 15 g Bacto agar per liter. In the case of phage titers, soft agar (7 g Bacto agar per liter) was used to overlay LB plates.

*Phages and titer methods*. A filamentous phage (f1) was used as the therapeutic virus (JB5 of Messenger, Molineux & Bull, 1999). As this virus establishes a lifelong infection of its host, we were motivated to engineer the virus to allow easy detection of infection and facilitate isolation of hosts known to be infected. An antibiotic resistance gene for kanamycin was cloned into the intergenic region, so that infected cells would grow in the presence of the antibiotic. As this phage was poor at forming plaques, it was titered with a sequential overlay method (Messenger, Molineux & Bull, 1999), as follows. Cells were mixed in soft agar and spread on agar plates to create a lawn; the suspension of f1 was streaked on top and allowed to dry. A second layer of soft agar was overlaid as a barrier. After 1–2 h of incubation at 37 °C, during which time infections occurred, a third layer of top agar with antibiotic was poured to limit further growth to cells carrying drug resistance. Infected cells form colonies in the first layer of soft agar. Kanamycin was only used to assay f1 phage titers and to generate cell stocks that were completely infected by f1; it was never used in dynamics assays because it would prevent growth of all but those cells already infected with f1.

The lytic RNA bacteriophage Q*β* was the lethal virus used to challenge and kill bacteria not protected by f1. Titers were determined as standard plaque-forming units.

### Dynamics assays

In all cases, cultures were incubated at 37 °C. Frozen cell stocks of A/*λ* were made by concentrating exponentially growing cells (grown in LB), aliquoting and freezing in 20% LB-glycerol at –80 °C. Cells were thawed just before use and added to 10 mL LB broth in 125 mL flasks, grown with aeration (170 rpm) for 60 min to a density of ∼10^8^ cells/ml, at which point phage were added. The volumes were diluted 10× when cell densities were high enough to inhibit further host growth (indicated in the figures). The time points for these dilutions were determined from preliminary trials based on the density of cells nearing 10^9^/mL if the culture was not diluted. The drug kanamycin, to which f1-infected cells were resistant, was never used in dynamics assays.

*Dynamics with both phages*. Cells were always grown for at least 1 h at 37 °C before phage addition. The timing and numbers of phage added are given in figure legends accompanying the results illustrated. Bacteriophage samples were taken at times indicated and cell densities were measured via plating on agar plates and incubating overnight at 37 °C. Samples of suspended f1 were purified by incubating the sample at 65 °C for 30–60 min to kill bacteria and kill Q*β*, spinning down debris and collecting the supernatant (tests confirmed that Q*β* was killed to at least 6 orders of magnitude by this procedure). Q*β* samples were purified by mixing the sample with chloroform to kill f1 phage and bacteria, centrifuging and collecting supernatant (chloroform is well known to kill f1, but we confirmed it nonetheless). Q*β* densities were measured from the number of clear plaques. Bacteriophage f1 titer was measured as described above. To determine the fraction of cells infected with f1 at each time point, colonies grown in the absence of drug were stabbed onto agar plates containing kanamycin. When the density of accompanying f1 is high at the initial plating (10^4^ or more per plate), this procedure allows some f1 infection of uninfected cells to occur during colony outgrowth, which would lead to an upward bias in the estimate of f1-infection and a downward bias in the estimate of uninfected cells. These rates were measured in controlled settings and found to have no meaningful effect on our dynamics estimates or conclusions.

*Dynamics with single phage*. Cells were grown (1–2 h) to a density of ∼10^8^ cells/ml and either f1 or Q*β* was added at a density as indicated. Each hour, phage were collected for titering and cells were plated on LB agar for density. In the case of f1 dynamics, overnight colonies from the LB agar plates were stabbed onto plates containing corresponding antibiotics as described above. Phage titers were determined as described above.

*Testing for cells resistant to* Q*β*. Using the colony-stabbing method to detect fractions of bacterial cells infected with f1, we observed instances of colonies that were not infected with f1 plated from cultures that had such a high density of Q*β* that all sensitive cells should have been killed (e.g., Fig. 7). We tested these colonies for Q*β* sensitivity by streaking them onto a fresh plate, across a zone of phage Q*β*. Streaks containing sensitive cells show a greatly depressed cell density at the intersection with Q*β* compared to streaks of resistant cells.

### Calculating therapeutic virus output from infected cells

The model requires a value of therapeutic virus output per infected cell. Estimating this parameter is complicated because, although the phage in a supernatant are easily counted, the cell density is not constant during the period of phage accumulation. A pair of differential equations enables us to describe the process:

(1) \begin{eqnarray*}{\dot {H}}_{v}={r}_{v}{H}_{v}\end{eqnarray*}

(2) \begin{eqnarray*}\dot {V}={b}_{v}{H}_{v}\end{eqnarray*}

where a superior dot indicates a derivative with respect to time, and notation is as in the dynamics equations presented later. Equation (1) is easily solved as

(3) \begin{eqnarray*}{H}_{v}(t)={H}_{v}(0){\mathrm{e}}^{{r}_{v}t}.\end{eqnarray*}

Equation (2) becomes

(4) \begin{eqnarray*}dV={b}_{v}{H}_{v}(0){\mathrm{e}}^{{r}_{v}t}dt,\end{eqnarray*}

(5) \begin{eqnarray*}V(t)=V(0)+{H}_{v}(0) \frac{{b}_{v}}{{r}_{v}} \left( {\mathrm{e}}^{{r}_{v}t}-1 \right) ,\end{eqnarray*}

(*dV* and *dt* in Eq. (4) are differentials). When measuring bacterial and phage titers at two time points, the only unknown in this equation is *b*_*v*_.

### T7 test of F plasmid presence

*E. coli* that otherwise support growth of the lytic phage T7 will abort T7 infections if they harbor the F plasmid (García & Molineux, 1995). To test for the presence of F in cells infected with f1, colonies grown in the absence of T7 were stabbed onto a plate with a high density of T7. Although T7 kills the cells that it contacts, it cannot produce progeny in those cells, so the stab from a colony will have enough cells to overwhelm the local T7 density and will regrow if those cells carry F. In contrast, a stab from cells that lack F will be extinguished by the T7 growth. For this test, we also confirmed that infection with f1 alone in the absence of F does not prevent T7 growth (using cells transfected with f1 DNA).

## Results

### Perspective: the relevant dynamics are short term

Half a century of phage biology has revealed that the co-culture of bacteriophage with a sensitive host leads to the eventual ascent of resistant bacteria, often to near complete recovery of the bacterial population as if no phage were present (Adams, 1959; Levin, Stewart & Chao, 1977; Bohannan & Lenski, 2000). In the early phases of this process, however, the bacterial population is killed down to the point that the only survivors are rare resistant individuals (who then reproduce and later restore the population). Any intervention to protect hosts should thus be evaluated above this intrinsic form of recovery. In particular, since the population will rebound in the long run even in the absence of an intervention, the relevant time frame to appraise the benefit of our system is short term, specifically (i) how much the intervention raises the minimum population size that survives the lethal phage epidemic, and (ii) how the population recovers from this nadir. In other applications, the benefit of an intervention may not be confined to the short term, but demonstrating a short term benefit ensures a long term benefit as well.

### A mathematical model

One of our motivations is to test the predictability of the empirical system. As host-parasite dynamics are intrinsically difficult to grasp intuitively, we use a mathematical model. The variable entities of our system are (i) a lethal parasite (density *L*) that, if unimpeded, will spread into the host population and cause high host mortality, (ii) a non-lethal, therapeutic virus (density *V*) that, upon infecting the host, protects it against subsequent infection or attack by the lethal parasite, (iii) uninfected hosts (density *H*_*u*_), and (iv) hosts infected with therapeutic virus (density *H*_*v*_). To specifically represent our empirical system, the model requires two additional properties of cells in this latter category: (v) cells transmit the therapeutic virus for life, and (vi) they transmit the infection to their progeny (vertical transmission).

As per standard assumptions, infection rates follow mass action, the number of hosts infected being the product of host density, viral density, and an adsorption rate parameter. In our system, the lethal virus is lytic, the infected host dying and releasing *b* viral progeny *T* minutes after infection (an index (*t* − *T*) indicates the value *T* minutes in the past); because lethal infections have such a short lifespan, an equation for hosts infected with the lethal virus is omitted. Our model assumes dynamics occur within a closed system, so we included a logistic growth function (*G*) to limit host density to a carrying capacity. Since f1 infection affects maximal host density and maximal host growth rate, we include separate logistic functions and growth rates for infected and uninfected hosts (Table 2).

The dynamics of this model system can be minimally described by the following system of differential equations:

(6) \begin{eqnarray*}\dot {V}=-{k}_{V}V{H}_{u}+{b}_{v}{H}_{v}\end{eqnarray*}

\begin{eqnarray*}\dot {L}=-{k}_{L}L{H}_{u}+{b}_{L}{k}_{L}L(t-T){H}_{u}(t-T) \end{eqnarray*}

\begin{eqnarray*}{\dot {H}}_{u}={r}_{u}{G}_{u}{H}_{u}-{k}_{v}V{H}_{u}-{k}_{L}L{H}_{u} \end{eqnarray*}

\begin{eqnarray*}{\dot {H}}_{v}={r}_{v}{G}_{v}{H}_{v}+{k}_{v}V{H}_{u} \end{eqnarray*}

where all parameters and variables are described in Table 2. In contrast to typical epidemiological models of infectious agents, we specifically account for the densities of free therapeutic virus and lethal virus, since our model system (using phages) allows us to monitor those densities. The model further assumes that upon infection with the therapeutic virus, protection is immediate and complete, which we know to be only approximately true (cf. Fig. 1). Possible extensions of this model are addressed in Discussion.

**Table 2:** Model variables and parameters.

Notation	Description	Values
Variables		
*H*_*u*_	Density of uninfected hosts	
*H*_*v*_	Density of hosts infected with therapeutic virus	
*V*	Density of therapeutic virus (free particles)	
*L*	Density of lethal virus (free particles)	
Functions		
*G*_*u*_	Density-adjusted growth factor for uninfected hosts (=1 − (*H*_*u*_ + *H*_*v*_)∕*C*_*u*_)	
*G*_*v*_	Density-adjusted growth factor for infected hosts (=1 − (*H*_*u*_ + *H*_*v*_)∕*C*_*v*_)	
Parameters		
*k*_*v*_	Adsorption rate of therapeutic virus to uninfected hosts (mL/min)	10^−10^
*k*_*L*_	Adsorption rate of lethal virus to uninfected hosts (mL/min)	10^−10^
*r*_*u*_	Growth rate of uninfected host (/min)	0.034
*r*_*v*_	Growth rate of hosts infected with therapeutic virus (/min)	0.027
*b*_*v*_	Rate at which hosts infected with therapeutic virus produce particles of the therapeutic virus (/min)	1
*b*_*L*_	Burst size of host infected with lethal virus	300
*T*	Time after infection that a host infected with the lethal virus dies and releases progeny (min)	45
*C*_*u*_	Carrying capacity for uninfected hosts (cells/mL)	2.8 × 10^9^
*C*_*v*_	Carrying capacity for uninfected hosts (cells/mL)	6.4 × 10^8^

Although it is not possible to solve the system of differential equations (6) directly, solving them numerically for particular parameter combinations is straightforward. All numerical solutions presented in the figures were obtained from Berkeley Madonna (version 9.0.123, with method Runge–Kutta 4 and a step size of 0.001), but graphical solutions from Mathematica (version 10.4.0.0) were used to verify the Madonna solutions. Mathematica files are included as supplements.

Runs illustrated below used the parameter values from Table 2. Some of those values are from direct estimates (*r*_*u*_, *r*_*v*_, *C*_*u*_, *C*_*v*_, from Table 1), others approximately so because of a highly variable estimate (*b*_*v*_ = 1). Our parameter values are within an order of magnitude of those of Wan & Goddard (2012), who obtained their estimates by curve fitting instead of direct measurement. Values assigned to adsorption rates and to Q*β* burst size and lysis time approximate values from the literature (Kim & Yin, 2004; Tsukada et al., 2009); it should be noted that Q*β* lysis time estimates from these sources vary from 40–60 min and burst size varies by an order of magnitude. However, all estimates (and the parameter values chosen here) allow for rapid Q*β* increase in the time frames and cell densities used here. Results illustrated in figures below that are sensitive to parameter values will be noted accordingly.

### Dynamics with single viruses

It is easily appreciated that the simplest and most straightforward dynamics occur when only one virus infects the host population. This case is of interest at least to enable a clear determination of model fit for each virus but also to ascertain general behaviors. To facilitate comprehension, we henceforth use ‘therapeutic virus’ for f1 and ‘lethal virus’ for Q*β*.

#### Therapeutic virus only

**Figure 2:**
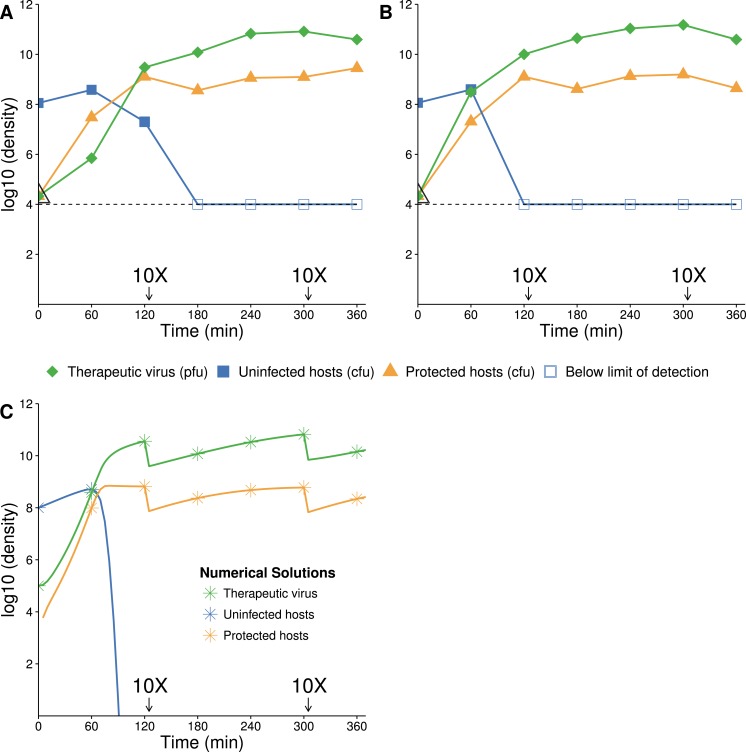
Growth dynamics of therapeutic virus on a high density of susceptible hosts. (A, B) Two assays of experimental densities of hosts and therapeutic virus (phage f1). Therapeutic virus (phage f1) was added to a culture of exponentially growing A/*λ* (≈10^8^ cells/mL) at a concentration of ≈2 × 10^4^ phage/mL. Host and phage densities were monitored each hour and 10X dilutions were made immediately after 120 and 300 min to avoid high densities that would limit cell growth and suitability as a host. The open black triangle at time 0 is an upper limit of therapeutic-virus infected hosts, under the assumption that all free therapeutic virus infects immediately. As such, it provides the highest possible value of the initial density of therapeutic-virus infected hosts and thus the lowest possible rate of therapeutic virus spread during the first hour. Note that the initial host density is not at carrying capacity, so some of the long term increase in therapeutic-virus infected hosts is by reproduction of protected hosts. The decline in uninfected hosts is slower than predicted, possibly for the same reason that Q*β* does not kill hosts to the predicted level. (C) Numerical analysis of therapeutic virus and host over time (parameter values are given in Table 2; initial values were 10^5^ phage/mL for free therapeutic virus, 10^8^ cells/mL for uninfected cells, and 0 cells/mL for therapeutic-virus infected cells). In contrast to the empirical results, virtually the entire host population is infected in 1.5 h. Results are broadly robust to variation in phage parameter values.

When therapeutic virus alone is introduced to the population, the model predicts a rapid spread by infection and no decrease in host density (except through a reduction in carrying capacity). Virtually the entire population becomes infected within 3 h (Figs. 2A and 2B). Total density does not decline because the therapeutic virus does not kill, it merely converts the state of the host. Numerical solutions support the observations at least qualitatively (Fig. 2C), and this outcome is broadly robust to wide variations in parameter values.

**Figure 3:**
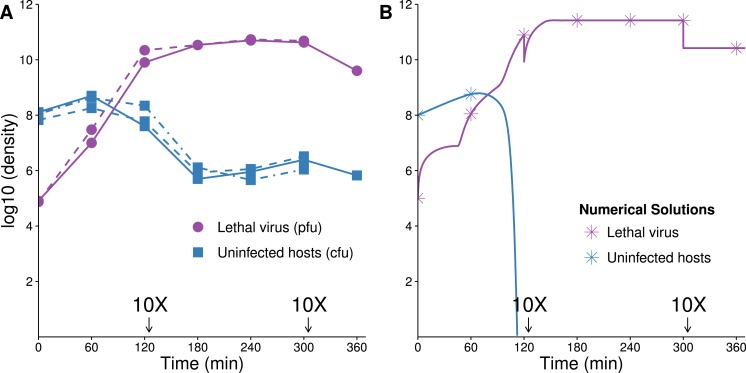
Growth dynamics of lethal virus (Q*β*) on an initially high density of susceptible hosts. (A) Three replicates of observed densities of host and phage over time (one replicate provides host density without phage density). Lethal virus was added to a culture of exponentially growing hosts (≈10^8^ cells/mL) at an initial density of ≈8 × 10^4^ phage/mL. Host density shows a far more shallow decline than expected from the numerical analyses (in B). 10× dilutions were made immediately after the indicated sampling. (B) Numerical analysis predicting densities of lethal virus and host shows a rapid loss of hosts—to orders of magnitude lower values than in empirical runs. Curves show the numerical output; for visual comparison, symbols are placed at the same times as in the empirical assays. 10× dilutions were imposed in the numerical analyses at the same times as in empirical assays. Results are broadly robust to phage parameter values. (See Table 2 for parameter values; initial values of variables were: lethal virus =10^5^ phage/mL, host =10^8^ cells/mL.)

#### Lethal virus only

The dynamics are expected to follow standard models for lytic phages (Levin, Stewart & Chao, 1977): introduction of the lethal virus into a population of susceptible hosts should be followed by a rapid and profound decline in host abundance. Our numerical analyses obey this prior result, showing a rapid increase in the phage density as the sensitive host population drops by 8 logs in ≈140 min (Fig. 3B). In a closed system, with no viral death or washout, the host population density should not rebound from this nadir except through evolution of hosts resistant to the phage. As our model does not allow any form of resistance to Q*β*, there is no recovery of the bacterial population.

The experimental dynamics when only the lethal virus is present exhibit a shallower decline of hosts than predicted; host density levels off and even starts to rise—in clear violation of the model (compare Fig. 3A with 3B). This resilience of the host population likely reflects a population of hosts that is initially variable in the level of phage receptor expression, followed by disproportionate viral killing of the most susceptible hosts and outgrowth of the least susceptible hosts (Lenski, 1988; Chapman-McQuiston & Wu, 2008a; Chapman-McQuiston & Wu, 2008b; Bull et al., 2014, see our Discussion). Despite the fact that the cells are derived from a fully susceptible parent, the variation arises too rapidly to be avoided, hence the phage kill the population down by only a couple logs.

### Dynamics with both viruses

#### Perspective

Even for a single set of parameter values, there are myriad conditions to consider when both viruses are introduced, depending on initial abundances and the time when each virus is introduced. Yet the intuitive expectation in all cases is that the population should ultimately be dominated by a high density of hosts infected with the therapeutic virus, because (i) infection with the therapeutic virus protects the host and all descendants from being killed, and (ii) the lethal virus is never lost from the population, so sensitive hosts are always at risk. How this endpoint is reached depends on which virus is introduced first, however. This outcome also requires that intrinsic host resistance is rare or absent in the initial population.

When the therapeutic virus invades first, the host population can be assumed to reside near its maximum density, because it has not been decimated by the lethal virus. Therapeutic virus spread will be largely infectious. In the extreme case, with initial host density near carrying capacity, the density of hosts may remain almost constant while their state changes from uninfected to infected by therapeutic virus, with little death and little reproduction by therapeutic-virus infected hosts. If protection against lethal virus is total and if the therapeutic virus is introduced well in advance of the lethal virus, this case reduces to that when only therapeutic virus is introduced, because the lethal virus has nothing to infect when finally introduced. Given that protected hosts support a low level of lethal virus growth (Fig. 1), the lethal virus is expected to invade but with little impact on host density.

The case when the therapeutic virus invades second is more complicated. To a suitable approximation for intuition, the effect of introducing the lethal virus first is to reduce host density prior to therapeutic virus introduction. Upon introduction of the therapeutic virus, there can be infectious spread, but the number of hosts gained through infection can be no more than the standing density of hosts–which will be several orders of magnitude less than carrying capacity. Henceforth, hosts infected with therapeutic virus will rise to carrying capacity by reproduction because both they and their progeny are protected from the lethal virus. This latter increase of therapeutic-virus infected hosts is slower than infectious transmission. Thus, we expect the infectious component (which is fast) to predominate when therapeutic virus is introduced first but the vertical component (which is slow) to predominate when therapeutic virus is introduced second. This latter expectation is strictly a consequence of the fact that protection is lifelong, and progeny of protected hosts are also protected.

#### Numerical solutions

**Figure 4:**
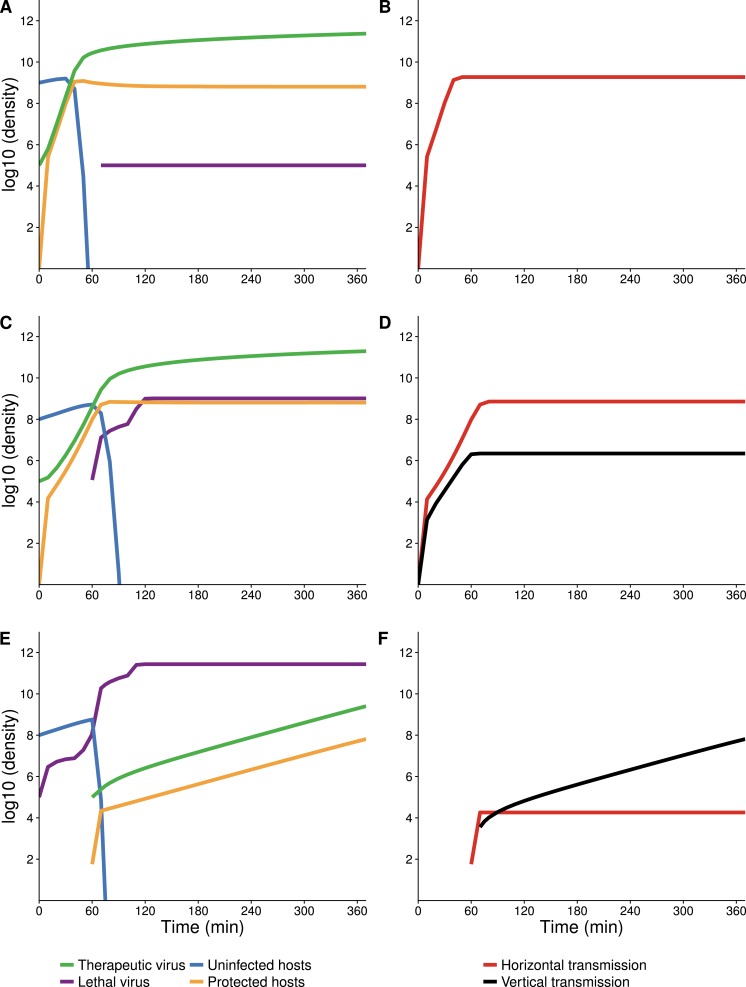
Numerical analyses show that the contribution of vertical versus horizontal transmission to therapeutic virus spread depends on initial density of hosts. Full dynamics are shown on the left, cumulative amounts of horizontal (orange) and vertical transmission (black) on the right. (A, B) Therapeutic virus is introduced first. The host density is at carrying capacity when the therapeutic virus is introduced, so there is a complete change from susceptible to resistant hosts without any change in density—all increase is horizontal (through infection). (C, D) Therapeutic virus is introduced first, but host density is below carrying capacity at the start. There is now a visible contribution to the increase in protected hosts from vertical transmission, although most spread is still horizontal. (E, F) Therapeutic virus is introduced second. The lethal virus reduces host density several logs by the time the therapeutic virus is introduced. There is then an initial period of infectious (horizontal) spread by the therapeutic virus on the remaining hosts, but within an hour, growth is mostly due to vertical transmission, and the vertical component would continue until the density of therapeutic-virus infected hosts increased another 2 logs beyond that shown—because host density is so low at this point. Although the details of each run are sensitive to parameter values, the basic behaviors illustrated here are robust. All virus introductions were at 10^5^ phage/mL at 0 or 60 min, as indicated. Initial (uninfected) host density was 10^9^ cells/mL in (A, B) and 10^8^ cells/mL in (C, D) and (E, F).

Numerical solutions of the system of differential equations (6) support intuition about the timing of different processes of therapeutic virus increase (Fig. 4). For therapeutic virus-first, when the sensitive host population is at carrying capacity, the conversion is almost entirely infectious (horizontal) (Figs. 4A and 4B). If the initial host population is modestly below carrying capacity, there is again rapid infectious spread, but now it is closely coupled with vertical spread; the relative amounts of each type of increase depend on how far below carrying capacity the initial population resides (Figs. 4C and 4D). In contrast, when therapeutic virus is introduced second, there is a brief infectious phase, but most subsequent growth to carrying capacity is through reproduction of infected hosts (vertical) (Figs. 4E and 4F). The latter process is so slow that host density is still 1–2 orders of magnitude below carrying capacity in the time frame shown.

**Figure 5:**
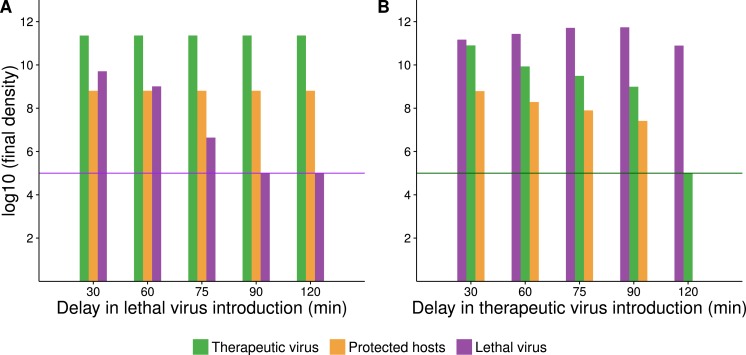
Numerical analyses show that the time between introduction of therapeutic virus and lethal virus affects the spread of the second virus. Bar height gives the final density of the respective phage or host (taken at 420 min after addition of the first virus). *X*-axis gives the time delay between first and second virus introduction. In all cases, initial starting host cell density was 10^8^ cells/mL. Both viruses were introduced at a density of 10^5^ phage/mL (indicated by a purple or green horizontal line for A and B respectively). (A) Endpoint densities when therapeutic virus is introduced first. Free therapeutic virus and therapeutic-virus infected hosts are largely unaffected because they are introduced early enough to substantially outrun the lethal virus. However, therapeutic virus impact in suppressing the lethal virus increases with the delay in introduction of the lethal virus. (B) Endpoint densities when therapeutic virus is introduced second. Now the head start of the lethal virus means that its density is largely unaffected by the therapeutic virus, but the impact of the therapeutic virus in protecting hosts declines with the delay in its introduction. Results of individual runs are quantitatively sensitive to parameter values, but the qualitative behavior illustrated is robust.

The delay between introduction of therapeutic virus and lethal virus is expected to influence dynamics up to a point (Fig. 5). In the therapeutic virus-first case, the lethal virus spread will depend on the number of unprotected hosts remaining at the time of its introduction, so a shorter lag between therapeutic virus and lethal virus introduction will promote more killing and higher densities of lethal virus. Likewise, when the therapeutic virus is introduced second, its spread will be greater the sooner it follows the lethal virus, because more hosts will be available to be infected by the therapeutic virus.

#### Empirical results: therapeutic virus first

**Figure 6:**
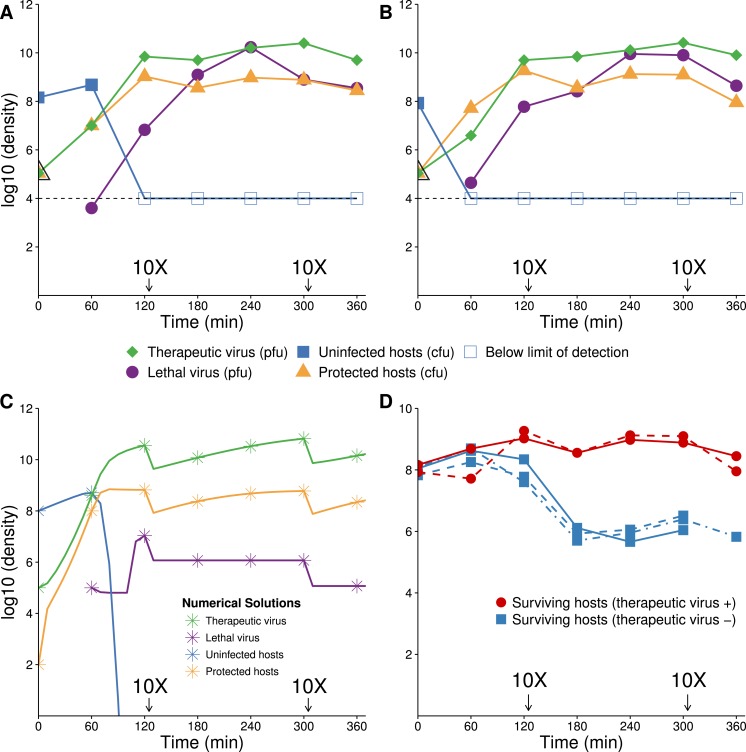
Growth dynamics when therapeutic virus is introduced before lethal virus. (A, B) Two replicates of observed experimental densities when therapeutic virus is introduced 60 min prior to lethal virus. Free therapeutic virus (≈10^5^ phage/ml) was added to a culture of growing hosts (≈10^8^ cells/ml). After 60 min of growth, lethal virus was added (at ≈4 × 10^3^ phage/ml). The open black triangle is an upper limit of therapeutic-virus infected hosts under the assumption that all free therapeutic virus infects immediately. As such, the slope shown for the yellow line (triangles) during the first hour is lower, perhaps much lower than the true slope. 10 dilutions were made immediately after the times indicated. (C) Numerical dynamics. 10× dilutions are introduced at the same times as in the empirical assays, and symbols are placed on the curves at the same times as samples were assayed empirically. Parameter values are from Table 2. (D) Comparison of the surviving host dynamics in response to lethal virus for a population into which therapeutic virus was introduced (circles, red) versus a population in which therapeutic virus was not introduced (squares, blue). Replicates are indicated as dashed vs. solid lines. The blue curves are from Fig. 3; the red are from (A) and (B) but combine protected and uninfected host densities.

Empirical results broadly agree with the numerical analyses (Fig. 6). Introduction of therapeutic virus first led to a rapid increase of hosts infected with the therapeutic virus. With a low initial ratio of therapeutic virus to cells (0.001) and the 1 h delay between therapeutic virus and lethal virus introductions, many uninfected hosts remained unprotected when the lethal virus was introduced; lethal virus density increased considerably (Figs. 6A and 6B). Nonetheless, the benefit of the therapeutic virus was evident from its huge effect on surviving host density (Fig. 6D).

**Figure 7:**
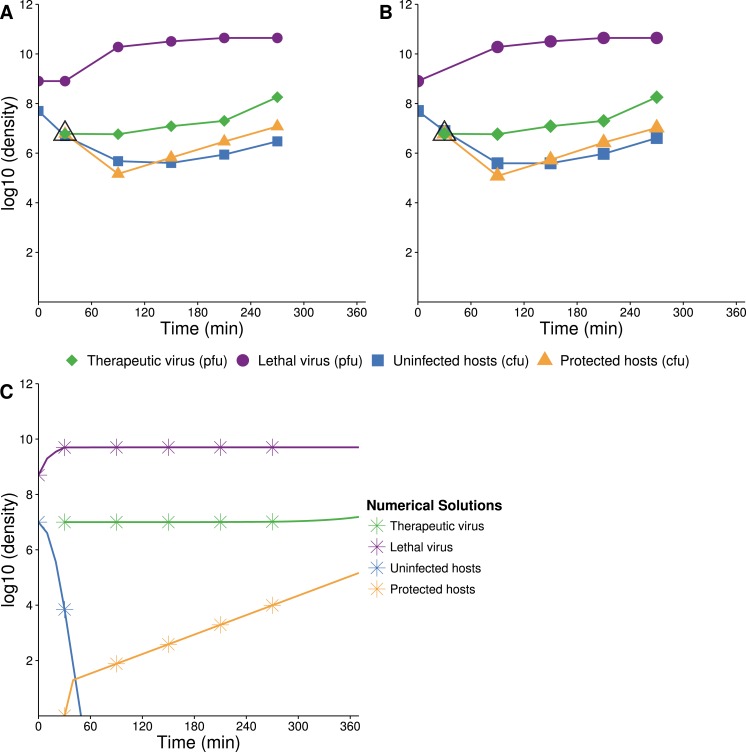
Growth dynamics when lethal virus is introduced first. (A, B) Experimental dynamics when lethal virus is introduced 30 min prior to therapeutic virus (two replicates). The increase in protected hosts (yellow triangles) is slow because it is by vertical transmission—reproduction of already-protected hosts. Lethal virus (4.4 × 10^8^ phage/ml) was added to a culture of hosts (8.8 × 10^6^ cells/ml). After 30 min, free therapeutic virus (7.1 × 10^6^ phage/ml) was added and densities were monitored over 4 h. The open black triangle is an upper limit of therapeutic-virus infected hosts, under the assumption that all free therapeutic virus virus infects immediately. (C) Numerical analyses illustrate that the biggest component of the increase in protected hosts comes from vertical transmission rather than horizontal transmission (the curve denoted with yellow stars; stars are placed at the same times as in the empirical assays). Initial conditions: lethal virus =5 × 10^8^ phage/ml, uninfected host =10^7^ cells/ml, therapeutic-virus infected host = 0 cells/ml, and free therapeutic virus =10^7^ phage/ml at 30 min.

One anomaly between the numerical analyses and empirical data was evident in the density of free lethal virus, which exceeded 10^10^/ml in culture compared to the predicted maximum of just over 10^6^. This suggests either that the density of therapeutic-virus infected cells increased more slowly than expected or that protection from the therapeutic virus was not immediate or only partial; the latter is supported by the observations in Fig. 1.

#### Empirical results: therapeutic virus second

The preferred design for this experiment would be to add lethal virus to a host population near carrying capacity and let the population crash before adding therapeutic virus. A technical problem with this approach is the rapid selection and rise of resistant bacteria even before introduction of the therapeutic virus. Our initial conditions were thus established by adding a low density of hosts to a high density of lethal virus, approximating the equilibrium that would have resulted after a high density host population crashed. Therapeutic virus was introduced 30 min later.

The empirical dynamics are in reasonable agreement with numerical analyses (Fig. 7). Within the first couple minutes of therapeutic virus introduction, there is an initial jump in density of therapeutic-virus infected cells (from 0). This initial rise is followed by a slower and steadier increase in density, with regression slope estimates of =1.45 and 1.48 (base *e*, per hour, using the last three samples in Figs. 7A and 7B respectively), statistically compatible with the spread by vertical transmission of infected hosts in the absence of lethal virus (Table 1).

There was an unanticipated persistence of therapeutic-virus-free hosts up to 4 h after therapeutic virus was introduced (nearly 20–30% were not carrying therapeutic virus, as evident from their lack of drug resistance). Those cells were plated and the resulting ∼18 h colonies were tested for lethal-virus sensitivity. It appeared that they were mostly resistant to the lethal virus, but a small subset appeared to be sensitive. However, these assays were difficult to interpret: the density of lethal virus was so high at plating that 18 h colonies commonly contained contaminating lethal virus. It is thus possible that colonies became exposed to lethal virus during their outgrowth and only then evolved resistance, in which case the count of resistance to the lethal virus was an overestimate. Even so, the presence of even a modest portion of sensitive cells in these populations is unexpected if they were fully susceptible to infection. We expect that they were partially resistant cells, as suggested previously for Q*β* (Lenski, 1988, and see Discussion).

### Blocking vertical transmission hampers the therapeutic virus’s effect

A unique property of our therapeutic virus (f1) is that the protection afforded to the host is transmitted vertically to the offspring, which is not necessarily true of all possible therapeutic viruses. This property is what allows the density of protected hosts to increase by reproduction, and thus for the population to rebound moderately fast after being suppressed by the lethal virus. We thus considered the effect of abolishing vertical transmission; as there is no known way to abolish vertical transmission of f1, we did so by numerical analysis. Equations (6) were modified so that the *r*_*v*_*G*_*v*_*H*_*v*_ term in the equation for ${\dot {H}}_{v}$ was moved to the equation for ${\dot {H}}_{u}$, so that all births from protected cells gave rise to uninfected, unprotected progeny.

**Figure 8:**
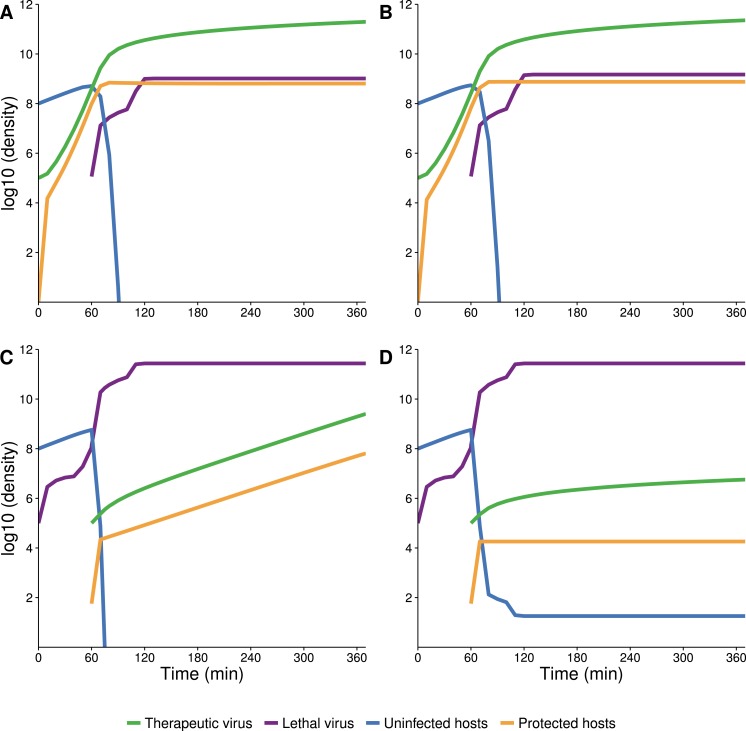
Numerical analyses showing the effect of vertical transmission to the increase in density of protected hosts. (A, B) Therapeutic virus introduced first with vertical transmission included (A) or blocked (B). Dynamics are indistinguishable due to nearly all increase in protected hosts coming from new infections (horizontal transmission). (C, D) Therapeutic virus introduced second with vertical transmission included (C) or blocked (D). Dynamics of protected hosts (orange triangles) are profoundly affected by blocking vertical transmission. Early on, dynamics are similar, as therapeutic-virus infected host densities rise quickly from horizontal transmission, until most hosts are infected. When vertical transmission is blocked, there is no further noticeable rise in therapeutic-virus infected hosts compared to the moderate rise observed when vertical transmission is included. Viruses were added at 10^5^ at times indicated. Initial host densities were 10^8^ cells/ml.

When therapeutic virus is introduced first (representative runs in Figs. 8A and 8B), the dynamics are almost indistinguishable whether vertical transmission is allowed or blocked. This near equivalence is due to the fact that nearly all increase in protected hosts is from infection, even when vertical transmission is allowed. In contrast, and also as expected, blocking vertical transmission of the therapeutic virus has a pronounced effect when the therapeutic virus is introduced second. The density of therapeutic-virus infected hosts rises quickly by horizontal transmission until nearly all hosts are infected (gold curves), but host density is initially well below carrying capacity. When protection is vertically transmitted, the population density rises moderately fast by reproduction (Fig. 8C). Yet when vertical transmission of the therapeutic virus is blocked, any further increase in hosts is almost negligible because most offspring are killed by the lethal virus (Fig. 8D).

## Discussion

We developed, modeled and tested a ‘virus wars’ system in which a transmissible, non-lethal virus (the therapeutic virus) protects its host from a lethal virus. Protection is by conferring resistance against the lethal virus, by affecting host gene expression rather than by genetically converting its host to a resistance state, although the dynamics are the same for either mechanism of protection.

Two motivations underlie our efforts. One is to suggest a new mechanism of dynamical suppression that, along with previously proposed mechanisms, might be used to assist the treatment of chronic infections such as HIV-1 (Schnell et al., 1997; Mebatsion et al., 1997; Weinberger, Schaffer & Arkin, 2003; Metzger, Lloyd-Smith & Weinberger, 2011; Ke & Lloyd-Smith, 2012). Our empirical system used bacteriophages instead of eukaryotic viruses, but the dynamics of phages are modeled in a similar way as for other types of dynamical suppression of HIV-1 (Weinberger, Schaffer & Arkin, 2003; Metzger, Lloyd-Smith & Weinberger, 2011, supplement). A second motivation is to compare predicted and observed dynamics to determine if it is generally feasible to predict dynamics with moderate knowledge of the biology. For this second goal, a parameterized, mathematical model was solved numerically and compared to experimental observations.

It is too early to identify the actual agents that might be used in an application against a human chronic infection such as HIV, and even if such transmissible agents were known, regulatory issues might thwart implementation in the near future. Our suggested approach is thus futuristic, but it is only a few steps from current practices. One current approach is to use a non-replicating viral vector to deliver gene therapy to stem cells *ex vivo* and then re-introduce the cells into the patient (Brown & Hirsch, 2015; Deal & Balazs, 2015; Schnepp & Johnson, 2015). The only requirement for our system is an agent that spreads among cells and converts them to a state that no longer allows infection or dissemination by the chronic virus. Such an agent could, in principle, be an attenuated derivative of a wild-type virus that blocks superinfection. The mechanism of blocking could stem from preventing infection (as in our study), to destroying the chronic virus on entry, to preventing the chronic virus from escaping. Genetic engineering may greatly facilitate the development of such vectors. Lentivirus vectors offer one class of possible implementations (Cockrell & Kafri, 2007; Metzger, Lloyd-Smith & Weinberger, 2011), although at present, use of autonomously replicating viruses has been extremely limited (Levine et al., 2006).

A general prediction of our empirical system is that, when the therapeutic virus and lethal virus are introduced into the same population, protected hosts should ultimately reach high frequency and dominate the population. In the empirical trials run, this was the observed behavior. The details of this take-over depended on initial conditions in an interesting way, however, on whether the therapeutic virus was introduced before or after the lethal virus. With the therapeutic virus introduced first (and with the host population dense), the protection spreads infectiously, with little change in overall host density. The hosts are merely changing their status from sensitive to resistant.

In contrast, when the lethal virus is introduced first, the population declines before protection is introduced. In this decimated host population, the therapeutic virus spreads infectiously at first. But as the available hosts become resistant to the lethal virus, the subsequent increase in protected hosts is by reproduction of the host population—a direct consequence of the therapeutic virus protecting not only the host cell that was infected but also protecting progeny. Simulations revealed that inheritance of the protected state is critically important to the success of the therapeutic virus when the therapeutic virus is introduced second to the lethal virus (Fig. 8).

Despite broad agreement between the predicted dynamics and the observed dynamics, some anomalies were noted. The main anomaly was that the host population did not behave as if all individuals were uniformly sensitive to viral infection. Consequently, the lethal virus did not suppress the population to nearly the level predicted, and the therapeutic virus did not infect as widely as predicted. It is likely that this anomaly is due to ‘phenotypic resistance,’ a widespread phenomenon in bacteria whereby the host population is variable in its sensitivity to infection (Lenski, 1988; Chapman-McQuiston & Wu, 2008a; Chapman-McQuiston & Wu, 2008b; Bull et al., 2014). In the case of a lethal phage, the phage will disproportionately kill cells that are more susceptible than others in the same population, the survivors transmitting the partial resistance state to their progeny and rebounding in number. The phenotypic resistance may result from from (stochastic) variation in gene expression for receptors, from variation in gene expression for molecules that block receptors, or even from gene expression induced in response to phage presence in the population (Bull et al., 2014). Phenotypic resistance has even been suggested from prior observations of short term dynamics with this phage (Q*β*) (Lenski, 1988). Some form of phenotypic resistance may well characterize most dynamical systems in which the host population is large.

Other anomalies between model and observation were also noted, such as the therapeutic virus not providing immediate, full protection and the therapeutic virus having a mildly deleterious effect on the host. Those are perhaps best classified as minor deviations from ideal behavior, easily accommodated by changes in parameter values or additional equations.

These anomalies point to several modifications in the model that would potentially increase its accuracy if appropriately parameterized. Equations could be added or modified to include (i) Q*β*-infected cells before they lyse, and (ii) delayed protection of f1-infected cells. Further extensions that could also be implemented include variation in sensitivity of the host, parameters dependent on host density and time since infection, reversible and irreversible adsorption steps, and loss of f1 infection. Many of these improvements would have quantitative but not qualitative effects, although variation in sensitivity of the host appears to have a significant effect on dynamics. The purpose of the paper was to start with a deliberately minimal model and see how well it matched empirical runs—as a test case for actual applications in the future. The simplified model appeared adequate to a first approximation in matching the data.

Although the mechanism of dynamical suppression studied here differs from those of other proposals, the overall success here of matching the models with empirical observations justifies continued consideration of dynamical suppression approaches. It is to be expected that every implementation will be unique in several ways and offer its own set of challenges. Yet there is reason to be confident that dynamical suppression mechanisms will be robust enough to lend themselves to broad classes of behaviors that can be captured with abstract models. There are even broader parallels, albeit shallow ones, between our models here and protection of a host population with a transmissible vaccine, a connection we develop elsewhere.

## Supplemental Information

10.7717/peerj.2166/supp-1 Supplemental Information 1 Supplemental data file

10.7717/peerj.2166/supp-2 Supplemental Information 2 Mathematica file for figure 6

10.7717/peerj.2166/supp-3 Supplemental Information 3 Mathematica file for Figs. 2, 3

10.7717/peerj.2166/supp-4 Supplemental Information 4 Mathematica file for dynamics without dilutions

## Additional Information and Declarations

### Data Availability

The following information was supplied regarding data availability: The raw data has been supplied as Data S1.
